# The impact of Chinese COVID-19 pandemic on the incidence of peripheral facial nerve paralysis after optimizing policies

**DOI:** 10.3389/fpubh.2023.1236985

**Published:** 2023-11-02

**Authors:** Erhui Yu, Fanyuan Jin, Wenhui Zhou, Junkang Chen, Huafeng Cai, Jinhua Hu, Lihua Xuan

**Affiliations:** ^1^The First School of Clinical Medicine, Zhejiang Chinese Medical University, Hangzhou, China; ^2^Department of Acupuncture and Moxibustion, Zhejiang Provincial Hospital of Chinese Medicine, Hangzhou, China

**Keywords:** peripheral facial nerve paralysis, COVID-19, China, Ramsay Hunt syndrome, H-B score

## Abstract

**Objective:**

To evaluate the impact of the COVID-19 pandemic on the occurrence of Peripheral Facial Nerve Paralysis (PFNP) in Chinese patients, identify contributing factors, and explore the relationship between COVID-19 and PFNP.

**Methods:**

We conducted a retrospective study covering the years 2020 to 2023, categorizing patients into three groups based on their visit dates: Group 1 (December 8, 2020 to February 28, 2021), Group 2 (December 8, 2021 to February 28, 2022), and Group 3 (December 8, 2022 to February 28, 2023). We collected and compared data on disease onset and patient characteristics among these groups.

**Results:**

In Group 3, following the widespread COVID-19 outbreak, there was a significant increase of 22.4 and 12.1% in PFNP cases compared to the same periods in the preceding 2 years (*p* < 0.001). Group 3 patients were more likely to be aged between 30 and 60 years, experience onset within 7 days, present with Ramsay Hunt syndrome, and have a higher H-B score of VI compared to the previous 2 years (*p* < 0.017). Logistic regression analysis revealed a strong association between the COVID-19 pandemic and the incidence of Ramsay Hunt syndrome in PFNP (OR = 3.30, 95% CI 1.81–6.03, *p* < 0.001).

**Conclusion:**

The incidence of PFNP increased in China after the COVID-19 pandemic, particularly in patients with Ramsay Hunt syndrome, indicating that COVID-19 infection can trigger and worsen PFNP.

## Introduction

Since the outbreak of COVID-19 in 2019, it has continued to disrupt people’s daily lives and become a major global public health problem in recent years (1). During the COVID-19 pandemic abroad, China has adhered to its “dynamic zero-COVID” policy for nearly 3 years (2), strictly implementing lockdowns and other active restrictive measures, resulting in much lower incidence, hospitalization, and mortality rates compared to other countries, and it did not have a large-scale epidemic (3, 4). However, on December 7, 2022, China optimized its epidemic policy and lifted restrictive measures (5). As a result, the Omicron variant quickly spread throughout the country (6), and the incidence of COVID-19 cases reached its peak.

Studies have shown that since the COVID-19 pandemic began worldwide, there has been an increase in the incidence of Peripheral Facial Nerve Paralysis (PFNP) (7), and some believe it is related to COVID-19 infection (8). COVID-19 known to be neuroinvasive and neurophilic (9), and related research has shown that abnormal neurological manifestations may be due to abnormal immune responses to COVID-19 (10). COVID-19 penetrates the brain through olfactory pathway, causing widespread tissue damage and severe inflammatory cascade response, accompanied by various neurological complications (11). Another hypothesis is the release of cytokines, including tumor necrosis factor-alpha (TNF-α), interferon-gamma (IFN-γ), interleukin-1beta (IL-1β), and interleukin-6 (IL-6). These cytokines can disrupt the blood–brain barrier (BBB) by activating the M1 phenotype of microglial cells, leading to rapid accumulation of insoluble toxic aggregates in different brain regions (12). Therefore, there is reason to consider the relationship between COVID-19 and PFNP.

Peripheral Facial Nerve Paralysis includes Bell’s palsy and Ramsay Hunt syndrome. There is limited literature on Ramsay Hunt syndrome, and we are the first to link COVID-19 with Ramsay Hunt syndrome. As far as we know, there has been no large-scale study reporting the incidence of PFNP in COVID-19 patients in China. Our study aims to analyze the changes in the incidence and clinical characteristics of PFNP patients after the large-scale COVID-19 virus infection in China, to determine whether there is a potential relationship between them.

## Research methodology

### Case collection and grouping

We retrospectively collected records of patients with PFNP as the main complaint at Zhejiang Provincial Hospital of Chinese Medicine from 2020 to 2023 using a computerized hospital system. The different years were divided into three groups. Group 1, Group 2, and Group 3 were patients with PFNP who visited our hospital between December 8, 2020 and February 28, 2021, December 8, 2021 and February 28, 2022, and December 8, 2022 and February 28, 2023, respectively.

### Diagnostic criteria

The main clinical manifestation is unilateral PFNP, with weakness on the affected side in closing the eyes, raising the eyebrows, puffing the cheeks, showing the teeth, and closing the lips, as well as tilting the corner of the mouth toward the opposite side. It may be accompanied by ipsilateral pain behind the ear or tenderness on the mastoid process. Depending on the location of facial nerve involvement, it may be accompanied by ipsilateral taste loss of the anterior two-thirds of the tongue, hyperacusis, and disorders of tear and salivary secretion.

### Inclusion and exclusion criteria

Included were patients with PFNP who met the diagnostic criteria, while patients with incomplete information and those with concomitant severe organic conditions such as cerebrovascular diseases, tumor-related illnesses, and hematological disorders were excluded.

### Statistical analysis

Quantitative variables are represented as mean (±SD) and *M(P_25_,P_75_)*, while categorical variables are reported as numbers (percentages). Statistical tests are all two-tailed, and *p* ≤ 0.05 is considered statistically significant. For quantitative data that follow a normal distribution, methods such as ANOVA and covariance are used. For unordered data, the chi-square test is used. Non-parametric rank-sum tests are used for quantitative data and ordinal data that do not follow a normal distribution. Multivariable quadratic logistic regression is used to explore the factors associated with the occurrence of PFNP after COVID-19.

## Results

### Comparison of the incidence rates of PFNP among the three groups

611 patients were diagnosed with PFNP for the first time in our hospital after a large-scale COVID-19 infection from December 8, 2022 to February 28, 2023, compared to 499 and 545 patients during the same period in the 2 years prior to the COVID-19 pandemic. The total number of cases in Group 3 significantly increased compared to Group 1 and Group 2 and this difference is statistically significant (*p* < 0.001), which indicates that the incidence of PFNP in our hospital increased by 22.4 and 12.1% compared to the previous 2 years in the same period.

### Comparison of the basic information among the three groups

In comparison with Group 1 and 2 (Table 1), Group 3 had a relatively higher number of patients between the ages of 30 and 60, and a relatively lower number of patients over 60, which was statistically significant (*p* < 0.017). The number of patients in Group 3 with an onset time of ≤7 days significantly increased (*p* < 0.017), while the number of patients with an onset time of ≥90 days decreased significantly (*p* < 0.017). However, there were no significant differences observed in terms of gender and affected side (*p* = 0.119, *p* = 0.611).

The number of patients classified as H-B III decreased significantly (*p* < 0.017), while the number of patients classified as Grade VI significantly increased (*p* < 0.017).

**Table 1:** Comparison of basic information among the three groups of patients with PFNP.

Variables		Group1	Group 2	Group 3	χ^2^	*p* value
Age	<30 years	135 (27.1)	144 (26.4)	150 (24.5)	11.98	0.017^*^
	30–60 years	284 (56.9)	315 (57.8)	396 (64.8)^**#**^		
	>60 years	80 (16.0)	86 (15.8)	65 (10.6) ^**#**^		
Gender	Male	233 (46.7)	269 (49.4)	323 (52.9)	4.26	0.119
	Female	266 (53.3)	276 (50.6)	288 (47.1)		
Site of involvement	Left	252 (50.5)	287 (52.7)	310 (50.7)	2.72	0.611
	Right	245 (49.1)	257 (47.2)	296 (48.2)		
	Bilateral	2 (0.4)	1 (0.2)	5 (0.8)		
Onset time	≥90 days	129 (25.9)	141 (25.9)	121 (19.8) ^#^	12.00	0.017^*^
	7–90 days	226 (45.3)	248 (45.5)	272 (44.5)		
	≤7 days	144 (28.9)	156 (28.6)	218 (35.7) ^#^		
Type of disease	Bell’s palsy	489 (98.0)	536 (98.3)	581 (95.1) ^#^	12.92	0.002^*^
	Ramsay Hunt syndrome	10 (2.0)	9 (1.7)	30 (4.9) ^#^		
H-B score	III	51 (10.2)	61 (11.2)	18 (2.9) ^#^	39.42	<0.001^*^
	IV	236 (47.3)	254 (46.6)	276 (45.2)		
	V	74 (14.8)	72 (13.2)	95 (15.5)		
	VI	138 (27.7)	158 (29.0)	222 (36.3)^#^		

^*^denotes comparison of the overall distribution of variables in the three groups, *p* < 0.05 is statistically significant; ^#^denotes group 3 compared with group 1 and group 2, *p* < 0.017 is statistically significant.

### The correlation degree of COVID-19 pandemic with factors related to PFNP

To further evaluate the correlation between the COVID-19 pandemic and factors related to PFNP incidence, a multiple logistic regression model (Table 2) was constructed with the COVID-19 pandemic as dependent variable and age, onset time, type of disease, H-B score as independent variables.

**Table 2:** The correlation degree of COVID-19 pandemic with factors related to PFNP.

Variables		β	Standard error of the coefficient β	Wald	P-value	OR	95% CI for OR	
							Lower	Upper
Age	<30 years^$^							
	30–60 years	0.24	0.12	3.73	0.054	1.27	1.00	1.61
	>60 years	−0.33	0.18	3.39	0.066	0.72	0.50	1.02
Onset time	≥90 days^$^							
	7–90 days	0.32	0.14	5.57	0.018^*^	1.38	1.06	1.80
	≤7 days	0.60	0.15	17.33	<0.001^*^	1.83	1.38	2.43
Type of disease	Bell’s palsy^$^							
	Ramsay Hunt syndrome	1.20	0.31	15.50	<0.001^*^	3.33	1.83	6.05
H-B score	III^$^-VI	0.28	0.05	28.77	<0.001^*^	1.33	1.20	1.47

^$^denotes control group; ^*^denotes *p* < 0.05 compared to control group associated with the onset of PFNP.

The results indicated that the COVID-19 pandemic was most strongly associated with the incidence of PFNP in patients with Hunt syndrome (OR = 3.30, 95% CI 1.81–6.03, *p* < 0.001).

## Discussion

On December 7, 2022, China optimized its COVID-19 prevention and control measures, leading to a significant increase in Omicron infections nationwide. Liu et al. (13) predicted that the peak of COVID-19 infections would occur between December 20 and 22, 2022. Therefore, during this period, researching the impact of COVID-19 virus infections on the incidence of PFNP is likely to yield reliable results.

Our research data show a significant surge in cases of PFNP following the COVID-19 pandemic in China compared to the preceding 2 years. This suggests an elevated incidence of PFNP after the COVID-19 pandemic. Similar findings have been observed in an increasing number of studies as well (8).

The study showed that the incidence of PFNP after COVID-19 pandemic primarily affected individuals in the 30–60 age group, with a decrease in the number of patients aged >60 years. This suggests that during the widespread COVID-19 infection period, younger patients are relatively more susceptible to the effects of PFNP. Supporting this hypothesis, a study on PFNP patients who visited the emergency department during the COVID-19 pandemic in Italy also found a similar pattern (14). Another reason could be that patients, both those who have had COVID-19 and those who have not, have concerns about worsening their condition by seeking medical care in hospitals (15). Older adult with underlying conditions such as hypertension and diabetes may choose to observe their symptoms at home instead of visiting hospitals for treatment.

Our study’s findings, which show a notable rise in patients with onset times of ≤7 days, are consistent with a study by Lima et al. In their study, they detailed the clinical features of eight COVID-19 patients who developed PFNP. In three of these cases, PFNP was the initial symptom, while in the other five patients, it manifested 2–10 days after other clinical symptoms began (16).

Patients with PFNP after the large-scale COVID-19 infection had higher H-B scores, possibly due to more severe neuronal damage caused by abnormal immune-mediated reactions in COVID-19 (17, 18). Additionally, even asymptomatic patients after COVID-19 infection can have an impact on the peripheral nerves (19).

Research indicates a strong link between the COVID-19 pandemic and Ramsay Hunt syndrome in cases of peripheral facial paralysis. Ramsay Hunt syndrome is primarily caused by the Varicella-Zoster Virus (VZV) (20), which establishes lifelong latent infections in the host’s neural ganglia (21). A study in Brazil noted an uptick in shingles cases during the COVID-19 pandemic (22), and research led by Joseph Katz and colleagues highlighted a significant correlation between VZV and COVID-19. This suggests that active replication or assembly of other viruses, such as COVID-19, may periodically reactivate VZV, potentially leading to Ramsay Hunt syndrome.

Research consistently highlights COVID-19’s significant impact on PFNP. Several studies increasingly suggest a link between COVID-19 and PFNP. Namavarian et al. (17) noted higher serum positivity among Bell’s palsy patients, while Islamoglu et al. (7) suggested PFNP could be the primary symptom of COVID-19. Furthermore, a study involving 348,088 COVID-19 cases identified 284 (0.08%) with PFNP, a notably higher rate compared to non-COVID-19 individuals (23).

Several mechanisms link COVID-19 to nerve damage in PFNP. First, COVID-19’s neurotropic nature can directly harm neurons (24), especially peripheral nerves that are more vulnerable than central nerves (25). This is consistent with findings of Khurshid et al. (16), which showed equal bilateral facial nerve involvement. Second, immune responses triggered by the virus can lead to nerve damage. COVID-19 may provoke autoimmune reactions against peripheral nerve myelin, causing demyelination (26, 27) and subsequent nerve damage. Third, facial nerve ischemia is a potential mechanism supported by observations of vascular changes in post-mortem studies of PFNP cases in COVID-19 patients (28–31). Lastly, reactivation of latent VZV is another possible mechanism (32). Clinical experience has linked various viruses, including HIV, mumps, and rubella, to PFNP (33).

## Conclusion

COVID-19 could exacerbate PFNP and has a more pronounced effect on Ramsay Hunt syndrome than Bell’s palsy. This study has relative limitations in terms of being a single-center study. Additionally, patients exhibit a certain degree of recall bias. But our study aids doctors in assessing virus-related PFNP during the COVID-19 and flu era. Clinical practitioners must remain vigilant about underlying peripheral nervous system disorders, like PFNP, which may worsen with severe respiratory viruses like COVID-19 or common conditions like the flu, preventing delayed or incorrect diagnoses. Future research will require larger sample sizes and animal experiments to establish causal relationships.

## Data availability statement

The original contributions presented in the study are included in the article/supplementary material, further inquiries can be directed to the corresponding author.

## Ethics statement

The studies involving humans were approved by First Affiliated Hospital of Zhejiang Chinese Medical University Ethics Committee (Approval Number: 2023-KL-193-01). The studies were conducted in accordance with the local legislation and institutional requirements. Written informed consent for participation was not required from the participants or the participants’ legal guardians/next of kin in accordance with the national legislation and institutional requirements.

## Author contributions

LX designed the study. EY contributed in drafting and editing the paper. FJ, WZ, JC, HC, and JH classified the data. All authors contributed to the article and approved the submitted version.
