# Corrigendum: The impact of Chinese COVID-19 pandemic on the incidence of peripheral facial nerve paralysis after optimizing policies

**DOI:** 10.3389/fpubh.2024.1522314

**Published:** 2024-11-26

**Authors:** Erhui Yu, Fanyuan Jin, Wenhui Zhou, Junkang Chen, Huafeng Cai, Jinhua Hu, Lihua Xuan

**Affiliations:** ^1^The First School of Clinical Medicine, Zhejiang Chinese Medical University, Hangzhou, China; ^2^Department of Acupuncture and Moxibustion, Zhejiang Provincial Hospital of Chinese Medicine, Hangzhou, China

**Keywords:** peripheral facial nerve paralysis, COVID-19, China, Ramsay Hunt syndrome, H-B score

In the published article, there was an error. Ramsay Hunt syndrome is a type of facial nerve paralysis, not Hunter Syndrome. This was a translation error. The disease previously stated was “Hunter Syndrome” throughout the article. The correct word is “Ramsay Hunt syndrome.”

The authors apologize for this error and state that this does not change the scientific conclusions of the article in any way. The original article has been updated.

